# Maternal–fetal platelet alloimmunization: Identification of risk factors for severe neonatal thrombocytopenia

**DOI:** 10.1111/trf.70234

**Published:** 2026-05-25

**Authors:** Elise Deroubaix, Marie‐Hélène Emektas, Gauthier Alluin, Pascale Renom, Fanny Rodermann, Mélissa Gilbert, Charles Garabedian, Véronique Houfflin‐Debarge

**Affiliations:** ^1^ CHU Lille, Pôle mère femme et nouveau‐né Lille France; ^2^ Laboratoire d'immunohématologie Etablissement français du sang Hauts‐de‐France‐Normandie Lille France; ^3^ Univ. Lille ULR 2694—METRICS: Évaluation des technologies de santé et des pratiques médicales Lille France

**Keywords:** anti‐platelet alloantibodies, fetal and neonatal alloimmune thrombocytopenia, fetal/neonatal intracranial hemorrhage, fetal/neonatal thrombocytopenia, intravenous polyvalent immunoglobulins

## Abstract

**Background:**

Feto‐maternal platelet alloimmunization (FNAIT) is a rare condition (1 in 1500 live pregnancies), most commonly diagnosed based on clinical neonatal hemorrhagic manifestations, which can vary in severity. The aim of our study was to identify risk factors for severe fetal/neonatal thrombocytopenia in cases of FNAIT.

**Materials and Methods:**

A retrospective study from 1993 to 2022 of pregnancies characterized by FNAIT. Data collected included patient characteristics, management of the pregnancies, and neonatal characteristics. Comparisons regarding pregnancy management and neonatal data were made between “index cases” and “subsequent pregnancies,” as well as between neonates with severe thrombocytopenia and those with no thrombocytopenia or moderate thrombocytopenia.

**Results:**

Seventy‐one patients were included in our study, corresponding to 119 pregnancies. Mothers of neonates with severe thrombocytopenia had a higher history of previous pregnancies characterized by severe neonatal thrombocytopenia (100.0% vs. 75.9%, *p* = .03). The FNAIT mainly involved the anti‐HPA‐1a system (75.0% vs. 54.5%, *p* = .02), and fewer treatments with IVIG were administered during pregnancy (6.6% vs. 34.1%, *p* < .001).

**Conclusion:**

FNAIT must be identified and appropriately managed to limit its potentially dramatic consequences, including fetal/neonatal death. Based on the patient's history, risk stratification for the occurrence of severe FNAIT in future pregnancies should be performed to tailor its management accordingly.

**Abbreviations**
AC: antibodies
ANSM: French National Agency for the Safety of Medicines and Health Products
EFS: French Blood Establishment
FDIU: fetal death in utero
FNAIT: fetal and neonatal alloimmune thrombocytopenia
GW: gestational weeks
HPA: human platelet antigen
ICH: intracranial hemorrhage
ITP: immune thrombocytopenic purpura
IVIG: intravenous polyvalent immunoglobulins
MTP: medical termination of pregnancy
TP: thrombocytopenia

## 1 INTRODUCTION

Fetal and neonatal alloimmune thrombocytopenia (FNAIT) affects approximately 1 in 1500 live pregnancies.^1^, ^2^ The diagnosis is often made during a first pregnancy (20%–59% of cases) based on neonatal thrombocytopenia or a fetal/neonatal hemorrhagic syndrome.^3^ FNAIT accounts for 3% of term neonatal thrombocytopenia cases (27% of severe neonatal thrombocytopenias),^4^, ^5^ which can lead to dramatic fetal or neonatal consequences, including intracranial hemorrhage (ICH) (affecting approximately 10%–20% of neonates in this context).^1^, ^6^Platelet alloimmunization occurs due to an incompatibility within the platelet system of the couple. There are multiple genetic polymorphisms within this system due to the presence or absence of specific antigens on platelets.^7^, ^8^, ^9^ During pregnancy, fetal and neonatal alloimmune thrombocytopenia occurs when fetal platelet antigens inherited from the father and different from those of the mother come into contact with the maternal immune system. If they are compatible with the maternal immune system, no antibodies are generated. If they are identified as alien unrecognized, it triggers the production of alloantibodies. These alloantibodies cross the placental barrier and bind to fetal platelets, leading to their destruction and causing fetal thrombocytopenia of varying severity.^9^, ^10^, ^11^

Certain risk factors for severe platelet alloimmunization have been identified. In particular, alloimmunization in the HPA‐1a system is associated with more severe thrombocytopenia and a higher incidence of intracranial hemorrhage (ICH).^12^, ^13^ The presence as well as high concentrations of anti‐HPA antibodies is strongly suggestive of severe neonatal alloimmune thrombocytopenia.^12^, ^14^ Moreover, HPA‐1b1b women carrying the HLA‐DRB3*01:01 antigen are thought to develop a stronger immune response within the platelet system.^13^

In a patient with a history of a pregnancy with severe fetal and neonatal alloimmune thrombocytopenia, intravenous polyvalent immunoglobulin (IVIG) treatment is currently recommended during pregnancy, at varying gestational ages and doses, depending on the patient's history and the medical team.^2^, ^7^ Management of subsequent pregnancies therefore depends on the risk factors for recurrence of severe thrombocytopenia.

The primary objective of our study was to identify risk factors for severe fetal and neonatal alloimmune thrombocytopenia.

## 2 MATERIALS AND METHODS

### 2.1 Population

This was a retrospective cohort study from January 1993 to December 2022. Patients who had undergone platelet immunology testing recorded in the database of the French Blood Establishment (EFS) Haut‐de‐France‐Normandie during or following a pregnancy were selected.

Obstetric data from patients with fetal and neonatal alloimmune thrombocytopenia were collected. The diagnosis of FNAIT was made when there was a platelet incompatibility within the couple or a fetus/neonate incompatible with the mother, associated with:

- the presence of platelet alloantibodies (even without suggestive clinical history),
- or neonatal thrombocytopenia with no other identified cause,
- or a medical termination of pregnancy due to cerebral hemorrhage with thrombocytopenia (with or without platelet alloantibodies), or without evidence of thrombocytopenia but with the presence of platelet alloantibodies.

Patients or pregnancies excluded were those whose collected data did not confirm or suggest platelet alloimmunization as the sole cause of thrombocytopenia or fetal/neonatal hemorrhagic syndrome, those for whom neonatal thrombocytopenia had a more probable cause than platelet alloimmunization, and those where the fetus/neonate was compatible with the mother (especially in subsequent pregnancies).This study was approved by the “Ethics Committee for Research in Obstetrics and Gynecology” (n°#2022‐OBS‐0402).

### 2.2 Maternal data and pregnancy data

Data were collected exhaustively, including patient characteristics, alloimmunization details, and pregnancy and neonatal characteristics.

Pregnancy, delivery, and neonatal data were compared between:

- The “index cases” group, which included first pregnancies that led to the diagnosis of FNAIT, and the “subsequent pregnancies” group, which included pregnancies following the “index case.”
- Neonates with severe thrombocytopenia (platelet count <50,000/mm^3^) or fetal/neonatal intracranial hemorrhage (ICH), and neonates with moderate thrombocytopenia (platelet count between 50,000/mm^3^ and 149.999/mm^3^) or with a normal platelet count (platelets ≥150,000/mm^3^).

### 2.3 Statistical analysis

Data are presented as median (1st and 3rd quartiles) with interquartile ranges for quantitative variables, and as frequencies and percentages for categorical variables. Comparisons of percentages were performed using bivariate analysis with the chi‐square test or Fisher's exact test when expected values were insufficient (*n* < 5) for categorical factors, and with the Mann–Whitney *U* test for quantitative factors. Statistical tests were performed with a bilateral significance threshold of 0.05. Data were analyzed using SAS software, version 9.4 (SAS Institute, Cary, NC).

## 3 RESULTS

### 3.1 Population description

**FIGURE A1:**
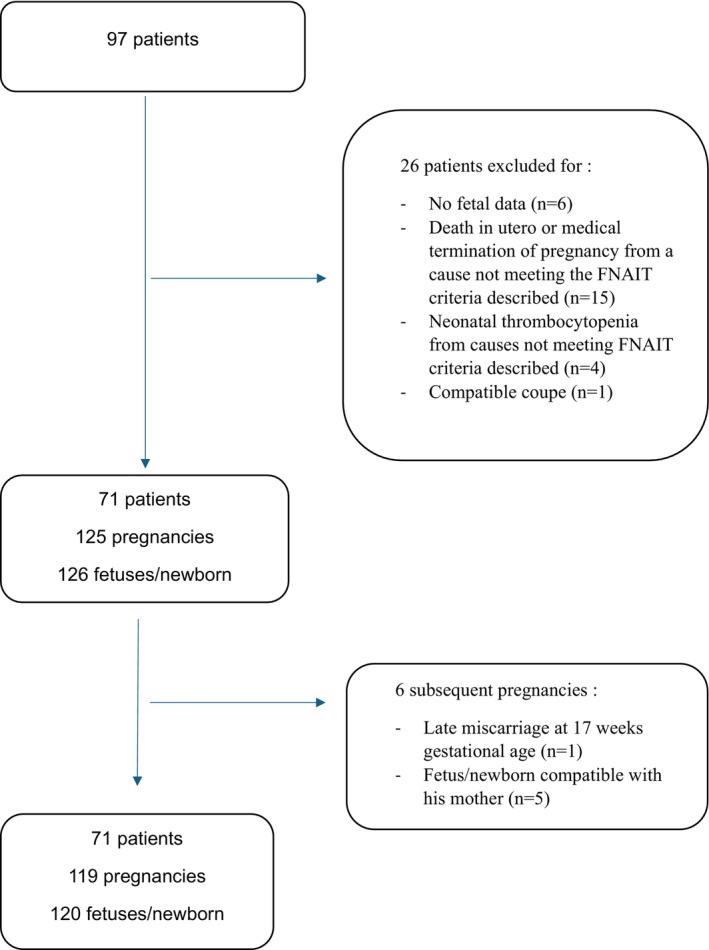
Flowchart.

Ninety‐seven patients were selected between January 1993 and December 2022. Among these patients, 26 were excluded for not meeting the inclusion criteria. Ultimately, 71 patients were included, corresponding to 125 pregnancies. One pregnancy was excluded due to a late miscarriage at 17 weeks of gestation with a non‐contributory fetal pathology analysis, and 5 pregnancies were excluded because the fetus/neonate was compatible with the mother. In total, 119 pregnancies, including 120 fetuses/neonates (one twin pregnancy in the “subsequent pregnancies” group), were included (Figure A1).

Twenty‐seven patients were primiparous (38.0%) at the time of diagnosis. Only one patient (1.4%) had a family history of platelet alloimmunization. Ten patients (14.1%) had a history of autoimmune disease, with no specific treatment for nine of them. One patient (1.4%) had a history of medical termination of pregnancy unrelated to FNAIT, and two patients (2.8%) had a history of peripartum death unrelated to medical termination of pregnancy (one due to a polymalformative syndrome without genetic testing information, and the other due to severe maternal–fetal infection) (Table 1).

**TABLE 1:** Characteristics of index case patients (*n* (%)).

Number of patients, *n* (%)	*n* = 71
Median age at first pregnancy characterized by fetal and neonatal alloimmune thrombocytopenia	28 [24–31]
Type of alloimmunization, *n* (%)	
HPA 1a	43 (60.6)
HPA 3a	4 (5.6)
HPA 5b	14 (19.7)
Associated with an ITP (*n* = 14)	3/14 (21.4)
HPA 5a	1 (1.4)
HPA 1b	2 (2.8)
HPA 3b	3 (4.2)
HPA 15a	2 (2.8)
HPA 15 b	2 (2.8)
Primiparous, *n* (%)	27 (38.0)
History of vascular‐placental pathology (pre‐eclampsia, retroplacental hematoma, HELLP syndrome), *n* (%)	2 (2.8)
Family history of platelet alloimmunization, *n* (%)	1 (1.4)
Personal history of autoimmune disease, *n* (%)	10 (14.1)
History of treatment for autoimmune disease, *n* (%)	1 (1.4)
History of medical termination of pregnancy, *n* (%)	1 (1.4)
History for death per partum (between 22 week gestational age28 days of life), *n* (%)	2 (2.8)
Spouse homozygous opposite, *n* (%)	53 (74.6)
Incompatibility within the couple, *n* (%)	71 (100.0)
Unique	35 (49.3)
Multiple	36 (50.7)
Cross match incompatible (*n* = 42), *n* (%)	39/42 (92.9)
Circumstance of discovery of alloimmunization, *n* (%)	
Fetal/neontal intracerebral hemorrhage (ICH)	15 (21.1)
Clinical signs other than ICH	47 (66.2)
Family history	1 (1.4)
Other^a^	4 (5.6)
Discovery before pregnancy	4 (5.6)
Presence of antiplatelet alloantibodies, *n* (%)	55 (77.5)
HPA 1a	36/55 (65.5)
HPA 3a	2/55 (3.6)
HPA 3b	1/55 (1.8)
HPA 5a	2/55 (3.6)
HPA 5b	11/55 (20.0)
HPA 15 a	1/55 (1.8)
HPA 15 b	1/55 (1.8)
HPA 1a et 5b	1/55 (1.8)
Number of patients with at least one pregnancy after index pregnancy, *n* (%)	33 (46.5)

^a^ Workup for maternal thrombocytopenia or autoimmune thrombocytopenic purpura discovered during pregnancy.

### 3.2 Fetal and neonatal alloimmune thrombocytopenia characterization

The diagnosis of fetal and neonatal alloimmune thrombocytopenia was made in 21.1% of cases based on the presence of fetal/neonatal intracranial hemorrhage. However, in two‐thirds of cases (66.2%), it was the presence of mild hemorrhagic signs in the neonate (petechiae, superficial body hematomas) that suggested the diagnosis. Less frequently, the diagnosis of platelet alloimmunization was made following platelet immunology testing, either after the discovery of maternal thrombocytopenia during pregnancy (in 5.6% of cases) or prior to pregnancy (due to a family history of FNAIT in a sister or following testing for maternal thrombocytopenia or von Willebrand disease, in 5.6% of cases) (Table 1).

The majority (60.6%) of alloimmunizations involved the HPA 1a system, followed by the HPA 5b system in nearly a quarter of cases (19.7%) and the HPA 3a system (5.6%). In 21.4% of cases, alloimmunization in the HPA 5b system was associated with immune thrombocytopenic purpura (ITP) in the mother. Alloantibodies were found in more than three‐quarters of patients (77.5%), with a strong predominance of anti‐HPA 1a antibodies (65.5%) (Table 1).

### 3.3 Management of subsequent pregnancies

Nearly half of the patients (46.5%) had at least one pregnancy following the index pregnancy (Table 1).

Anti‐HPA1a alloantibodies were identified in two‐thirds of cases (Table 1) and measured in 16.7% of cases (Table 2). One‐third of pregnancies (33.3%) were treated with intravenous polyvalent immunoglobulins (IVIG) at a dose of 1 mg/kg, starting treatment around 22 weeks of gestation (20 weeks – 24 weeks). Almost all of these pregnancies were from patients with a history of severe fetal and neonatal alloimmune thrombocytopenia in a previous pregnancy. One patient was treated due to severe maternal thrombocytopenia in the context of immune thrombocytopenic purpura. Five cases (31.3%) had complications following this treatment during pregnancy, of which 4 (80.0%) did not require hospitalization (including one case of flu‐like syndrome, one case of a skin rash, one case of chest pain, and one case of phlebitis), but a change in medication was required. In one case, hospitalization was necessary due to vomiting and diarrhea associated with electrolyte disturbances (Table 2).

**TABLE 2:** Management of subsequent pregnancies and postnatal characteristics.

	Total (*n* = 119)	Index case (*n* = 71)	Subsequent pregnancies (*n* = 48)	*p* ^a^
AC anti HPA 1a during pregnancy, *n* (%)	7/79 (8.9)	1/43 (2.3)	6/36 (16.7)	.04
Median AC level at start of assay (UI/l)	7 [9–16]	7	12 [9–16]	
Gestational age at 1st dosage (gestational weeks)	14 + 3 [11 + 0–19 + 6]	35 SA	14 + 3 [12 + 3–18 + 3]	
AC levels before IVIG treatment (UI/l)	12.5 [8.25–19]	7	16 [9–20]	
Corticosteroid therapy for maternal indication, *n* (%)	4 (3.4)	1 (1.4)	3 (6.3)	.30
IVIG treatment, *n* (%)	20 (16.8)	4 (5.6)	16 (33.3)	<.001
Indications, *n* (%)				
Family history	1/20 (5.0)	1/4 (25.0)	–	
History of severe maternal ITP in the context of ITP	3/20 (15.0)	2/4 (50.0)	1/16 (6.3)	.09
History of severe thrombocytopenia or ICH	15/20 (75.0)	–	15/16 (93.8)	
Fetal cerebral hemorrhage	1/20 (5.0)	1/4 (25.0)	–	
Median gestational age at treatment initiation (gestationnal weeks)	23 [20–24 + 1]	35 [29–35 + 4]	22 [20–24]	.16
Complications IVIG, *n* (%)	5/20 (25.0)	–	5/16 (31.3)	
With infusion stop	4/5 (80.0)	–	4/5 (80.0)	
With hospitalization	1/5 (20.0)	–	1/5 (20.0)	
ICH on antenatal imaging, *n* (%)	18 (15.1)	13 (18.3)	5 (10.4)	.30
Median gestational age of ultrasound (weeks gestational age)	32 + 4 [27 + 0–33 + 5]	32 + 6 [28 + 1–33 + 5]	32 + 0 [27 + 0–32 + 4]	.84
Newborn alive at birth, *n* (%)	108/120 (90.0)	61 (85.9)	47/49^b^ (95.9)	.11
Thrombocytopenia, *n* (%)	96/120 (80.0)	66 (93.0)	30/49^b^ (61.2)	<.001
Severe (<50,000/mm^3^ ou HIC)	76/96 (79.2)	57/66 (86.4)	19/30 (63.3)	.01
Moderate (50,000/mm^3^–149,999/mm^3^)	20/96 (20.8)	9/66 (13.6)	11/30 (36.6)	
Nadir platelets at birth, *n* (%)				
<30,000/mm^3^	53/107 (49.5)	37/61 (60.7)	16/46 (34.8)	.01
30,000/mm^3^–49,999/mm^3^	11/107 (10.3)	10/61 (16.4)	1/46 (2.1)	.02
50,000/mm^3^–149,999/mm^3^	20/107 (18.7)	9/61 (14.8)	11/46 (23.9)	.32
≥150,000/mm^3^	24/107 (22.4)	5/61 (8.2)	19/46 (41.3)	<.001
Hemorrhagic syndrome at birth, *n* (%)	69/120 (57.5)	58 (81.6)	11/49^b^ (22.4)	<.001
Petechiae	54/69 (78.3)	48/58 (82.8)	6/11 (54.5)	.05
ICH	20/69 (29.0)	15/58 (25.9)	5/11 (45.5)	.28
Superficial hematoma	14/69 (20.3)	9/58 (15.5)	5/11 (45.5)	.04
Other	1/69 (1.5)	1/58 (1.7)	–	
Hospitalization in neonatal intensive care unit, *n* (%)	40/63 (63.5)	29/36 (80.6)	11/27 (40.7)	<.001
Hospitalization in neonatal reanimation, *n* (%)	12/63 (19.0)	7/36 (19.4)	5/27 (18.5)	>.99
Related to FNAIT, *n* (%)	48/52 (92.3)	33/36 (91.6)	15/16 (93.8)	>.99

^a^ Comparison between “index cases” and “subsequent pregnancies.”

^b^ *n* = 49 because a pair of twins but same pregnancy.

Brain abnormalities were detected on ultrasound in 5 fetuses from subsequent pregnancies (10.4%). These included hydrocephalus, ventriculomegaly, or isolated intraventricular hemorrhages or those associated with intraparenchymal hemorrhages. These patients had a history of severe feto‐maternal platelet alloimmunization, but testing had not been performed during the first pregnancy (due to the couple's refusal after medical termination of pregnancy) or testing had been performed but was unknown to the team managing the subsequent pregnancy (Table 2).

### 3.4 Postnatal characteristics

A large majority of neonates were thrombocytopenic (80.0%), but significantly fewer in the “subsequent pregnancies” group compared to the “index cases” group (61.2% vs. 93.0%, *p* < .001), and with fewer severe thrombocytopenias or intracranial hemorrhages (63.3% vs. 86.4%, *p* = .01). Neonates from subsequent pregnancies had fewer hemorrhagic syndromes at birth (22.4% vs. 81.6%, *p* < .001). Neonatal intensive care unit (NICU) admissions were less frequent in the “subsequent pregnancies” group (40.7% vs. 80.6%, *p* < .001; Table 2).

### 3.5 Risk factors for severe thrombocytopenia

In cases of anti‐HPA 1a alloimmunization, more than two‐thirds of neonates (70.4%) had severe thrombocytopenia or neonatal ICH. In contrast, in cases of anti‐HPA 5b alloimmunization, more than half (63.2%) were non‐thrombocytopenic or had moderate thrombocytopenia. Less than 10% of severe thrombocytopenia cases were related to the HPA 5b system. Among HPA 5b alloimmunizations, 21.1% were associated with immune thrombocytopenic purpura (ITP), and all of these cases of HPA 5b alloimmunization with ITP were in the non‐thrombocytopenic or moderate thrombocytopenia group (Table 3).

**TABLE 3:** Comparison of newborns with severe thrombocytopenia versus moderate thrombocytopenia/non‐thrombocytopenia.

	Severe thrombocytopenia or ICH (*n* = 76)	Moderate thrombocytopenia or normal platelets (*n* = 44)	*p*
Circumstances of discovery of alloimmunization, *n* (%)			
Fetal ICH history	7/20 (35.0)	5/29 (17.2)	.19
History of neonatal thrombocytopenia	13/20 (65.0)	24/29 (82.8)	
History of severe neonatal thrombocytopenia, *n* (%)	20/20 (100.0)	22/29 (75.9)	.03
Last pregnancy (months)	34.7 [22.5–36.75]	34 [24–48]	.94
Type of alloimmunization, *n* (%)			
HPA 1 a	57 (75.0)	24 (54.5)	.02
HPA 3 a	4 (5.2)	4 (9.1)	.46
HPA 5 b	7 (9.2)	12 (27.3)	.02
Associated with an ITP	–	4/12 (33.3)	–
HPA 15 a	3 (3.9)	–	–
HPA 3 b	1 (1.3)	2 (4.5)	.55
HPA 5a	1 (1.3)	1 (2.3)	>.99
HPA 15b	1 (1.3)	1 (2.3)	>.99
HPA 1b	2 (2.6)	–	–
Cross match incompatible	42/45 (93.3)	17/21 (81.0)	.20
IVIG treatment during pregnancy	5 (6.6)	15 (34.1)	<.001
Median gestational age at start of IVIG treatment	21 + 5 [21 + 1–25 + 6]	21 + 5 [19 + 5–23 + 5]	.97
Brain damage on imaging	16 (21.0)	–	
Medical interruption pregnancy for brain damage	5 (6.6)	–	

Among the 20 pregnancies treated with IVIG, three‐quarters of the fetuses/neonates did not have severe thrombocytopenia (75.0% vs. 25.0%, *p* = .01).

In total, neonates with severe thrombocytopenia were more frequently born to pregnancies with a history of severe neonatal thrombocytopenia (100.0% vs. 75.9%, *p* = .03), HPA 1a system alloimmunization (75.0% vs. 54.5%, *p* = .02), and were less frequently treated with IVIG during pregnancy (6.6% vs. 34.1%, *p* < .001; Table 3).

## 4 DISCUSSION

Our retrospective study of 71 patients, involving 119 pregnancies corresponding to 120 fetuses/newborns, showed that in the context of fetal and neonatal alloimmune thrombocytopenia, newborns had more severe thrombocytopenias in cases of HPA 1a alloimmunization, a history of pregnancy with severe neonatal thrombocytopenia, and the absence of treatment with intravenous polyvalent immunoglobulins during pregnancy.

In our study, the circumstances leading to the discovery of fetal–maternal platelet alloimmunization were mainly mild neonatal clinical signs. Yet, in nearly a quarter of the cases, the presence of fetal/neonatal intracranial hemorrhage (ICH) guided the diagnosis.

Anti‐HPA 1a fetal–maternal alloimmunization was found to be the most frequent, which is consistent with the literature.^7^, ^12^ In fact, in a series reporting 1162 cases of neonatal platelet alloimmunization, 79% were anti‐HPA 1a alloimmunizations and 8%–9% were anti‐HPA 3a alloimmunizations.^3^ Within the literature, the HPA 5b system would account for 10% of FNAIT^1^; in our study, the HPA 5b system accounted for 20% immunizations. The discovery of other platelet systems allowed for the identification of other types of HPA, the frequency of which varies by country. Indeed, 95% of confirmed FNAIT are due to the HPA systems 1, 2, 3, 5, or 15, which are more or less immunogenic depending on ethnic origins; the HPA 9b, 4b, 6b, and 21b systems seem less immunogenic in Caucasians.^7^

The main risk of this immunogenicity is the occurrence of neonatal thrombocytopenia, which may be more or less severe. The first risk factor for severe thrombocytopenia identified in our study was HPA 1a system immunization. In our series of subsequent pregnancies, newborns with anti‐HPA 1a alloimmunization more frequently had severe thrombocytopenia or ICH (70.4% in cases of HPA 1a immunization). This pattern is consistently reported in the literature with around 90% of FNAIT cases associated with ICH attributable to the HPA 1a system.

Furthermore, in case of HPA 1a incompatibility, one‐third of fetuses present with thrombocytopenia below 50,000/mm^3^.^7^, ^15^ On the other hand, when considering HPA 5b immunization, fewer severe thrombocytopenias were found (about 15%).^16^ However, in our series, among the HPA 5b immunization, 36.8% of fetuses/newborns had severe thrombocytopenia. The HPA 3a system seems to cause alloimmunizations as severe as the HPA 1a system.^16^ In our study, only three cases of HPA 3a immunization were found, which do not allow us to draw a conclusion.

In three‐quarters of the patients in our series, alloantibodies were detected, with nearly two‐thirds of cases having anti‐HPA 1a alloantibodies. The absence of alloantibody detection in the case of a clinical picture suggestive of FNAIT and platelet incompatibility in the couple has been observed by several authors, with a variable frequency from 37%^3^ to 80%.^17^ This can be explained by the fact that current techniques remain poorly sensitive and do not allow for the detection of certain alloantibodies (e.g., fragile epitopes that are difficult to detect for the HPA 2 and 3 systems).^3^, ^17^, ^18^, ^19^ According to some authors, detecting alloantibodies early in pregnancy would be a sign of FNAIT severity,^3^, ^12^, ^20^ indicating the need for earlier treatment. These data were not studied in our series.

To predict the severity of FNAIT, some authors use the measurement of alloantibodies as a marker for thrombocytopenia severity. In the context of FNAIT, only anti‐HPA 1a alloantibodies are routinely measured. In our study, 8.9% of cases tested positive for anti‐HPA 1a antibodies, as other types of alloantibodies cannot currently be quantified with available techniques. Bertrand et al. demonstrated a link between a high level (above 28 IU/L) of anti‐HPA 1a antibodies measured at 28 weeks of gestation before treatment with IVIG and the existence of severe fetal/neonatal thrombocytopenia.^21^ However, other authors suggest that for patients whose FNAIT has already been diagnosed, measuring anti‐HPA 1a antibodies does not seem to predict the severity of thrombocytopenia. In fact, the level of alloantibodies varies during pregnancy, and the concentration tends to rise quickly before delivery.^19^, ^22^ Thus, there is not enough data currently to conclude on the prognostic value of alloantibody measurement during pregnancy.

The second risk factor for severe neonatal thrombocytopenia found in our study was a history of a fetus/newborn with severe thrombocytopenia or ICH in a previous pregnancy. Indeed, all newborns with severe thrombocytopenia in subsequent pregnancies had a history of severe neonatal thrombocytopenia. Among these cases, one‐third had a prior history of fetal or neonatal ICH. The literature also supports this, and several authors argue that patients with a history of severe FNAIT should be closely monitored, as they present a risk for the new pregnancy of severe neonatal thrombocytopenia^1^, ^7^, ^8^, ^16^, ^23^, ^24^; the recurrence rate of ICH could be as high as 80% in the absence of treatment.^1^, ^6^, ^8^, ^16^, ^23^, ^24^ Another risk factor for severe FNAIT could be the early gestational age at which ICH occurred in a previous pregnancy.^6^, ^16^ This aspect was not analyzed in our study.

In our series, the third risk factor for severe neonatal thrombocytopenia was the absence of antenatal IVIG treatment. This treatment was implemented in one‐third of subsequent pregnancies, and newborns from these pregnancies had fewer severe thrombocytopenias (6.6% of severe thrombocytopenia or ICH versus 34.1%). This treatment seems promising since preliminary studies have shown an increase in platelet count with IVIG treatment. In fact, in a pilot study in 1988, Bussel et al. showed that the administration of intravenous polyvalent immunoglobulins during pregnancy increased fetal platelet count (average increase of 72,500/mm^3^) and reduced the recurrence rate of ICH in patients with a history of severe FNAIT.^25^ Subsequently, many studies have discussed IVIG treatment during pregnancy as the only effective treatment for reducing neonatal thrombocytopenia.^6^ However, other authors are more cautious about the effects of antenatal IVIG treatment. In approximately two‐thirds of cases, women with a history of severe anti‐HPA 1a FNAIT in a previous pregnancy exhibited a stabilization or even a spontaneous rise in platelet counts.^26^

Currently, there is consensus on antenatal treatment for severe FNAIT, particularly those with a history of fetal/neonatal ICH.^13^, ^27^ However, for less severe FNAIT, protocols regarding the indication for antenatal IVIG treatment and its dosage vary by team. Randomized controlled trials comparing different IVIG doses exist, but none compare antenatal IVIG treatment with a placebo.^28^, ^29^

This treatment is debated due to several factors. First, intravenous polyvalent immunoglobulins are blood‐derived products, and thus, there is a real supply tension. Furthermore, their financial cost is significant, and it does not seem rational to use them for all patients with a history of FNAIT, regardless of severity. Finally, IVIGs have potential side effects, some of which can be severe, although few complications have been reported in our series. This is also true in the literature, where this treatment is considered safe and less dangerous than invasive procedures (in utero transfusion).^6^, ^24^, ^30^ Arnold et al. reported a low incidence of side effects from IVIGs: 10% of cases involved fever, and rare cases of chest pain, blood pressure abnormalities, bronchospasm, laryngeal oedema, hemolysis, acute renal failure, and aseptic meningitis.^20^

Decisions regarding preventive treatment should therefore be based on a risk stratification for severe thrombocytopenia according to the patient's history, and possibly, for HPA 1a FNAIT, based on the quantification of alloantibodies.^1^, ^6^, ^7^, ^15^, ^20^, ^30^ In France, the National Agency for the Safety of Medicines and Health Products (ANSM) considers anti‐HPA 1a platelet alloimmunization with a proven history of neonatal thrombocytopenia as a priority indication.^31^

Our study is limited by its retrospective design, with cases spanning approximately 30 years. During this period, practices evolved, notably in methods for investigating different platelet systems, the identification of new platelet alloantigens, the quantification of anti‐HPA 1a alloantibodies, and protocols for preventive IVIG therapy. Another limitation is the small number of cases, which reflects both changes in clinical practice and the rarity of this condition. Finally, our inclusion criteria were primarily based on symptomatic cases, enabling the identification of risk factors for severe outcomes; however, it is likely that more asymptomatic cases exist than those captured in our study. This also accounts for the higher frequency of alloantibodies observed compared to reports in the literature.

In our study, the risk factors for severe fetal/neonatal thrombocytopenia, and thus intracranial hemorrhage, were anti‐HPA 1a alloimmunization, a history of pregnancy with severe platelet alloimmunization, and the absence of preventive antenatal treatment with intravenous polyvalent immunoglobulins.

Due to the complexity and potential severity of FNAIT, all fetal or neonatal thrombocytopenias should be explored, and patients should be followed by a specialized team. A postnatal or preconception consultation should be offered to inform the couple clearly and understandably about FNAIT and define the management strategy for future pregnancies. In some countries, a flyer on fetal and neonatal alloimmune thrombocytopenia is given to patients, and multidisciplinary meetings are organized. Informing the couple helps raise awareness of the need for early monitoring in a specialized center for FNAIT during future pregnancies. Management of subsequent pregnancies (preventive treatment, monitoring, mode of delivery) will depend on the stratification of severe thrombocytopenia risk.

Given the need to deepen our knowledge and improve the management of patients with FNAIT, other preventive treatment options are being explored. An international multicenter randomized interventional study called FREESIA is currently underway to evaluate the effectiveness of a new human monoclonal Immunoglobulin G, NIPOCALIMAB, in reducing the risk of FNAIT. This is a double‐blind, placebo‐controlled study. Nipocalimab blocks the binding of IgG to the neonatal receptor for the crystallizable fragment of IgG, thereby reducing the active transfer of maternal humoral immunity to the fetus and preventing the passage of alloantibodies, thus preventing the occurrence of fetal thrombocytopenia.

## CONFLICT OF INTEREST STATEMENT

The authors have disclosed no conflicts of interest.

## Data Availability

The data that support the findings of this study are available in Pubmed at https://pubmed.ncbi.nlm.nih.gov/. These data were derived from the following resources available in the public domain: Pubmed, https://pubmed.ncbi.nlm.nih.gov/.
